# Case report: Self-expanding transcatheter valve implantation (Acurate Neo 2) in a very small native aortic annulus

**DOI:** 10.3389/fcvm.2023.1195486

**Published:** 2023-09-15

**Authors:** Massimo Medda, Francesco Casilli, Marta Bande, Maurizio Tespili, Francesco Donatelli

**Affiliations:** ^1^ Clinical and Interventional Cardiology Unit, Cardio-Thoracic Center, IRCCS Ospedale Galeazzi-Sant’Ambrogio, Gruppo San Donato, Milan, Italy; ^2^ Clinical and Interventional Cardiology Unit, Cardio-Thoracic Center, Istituto Clinico Sant’Ambrogio, Gruppo San Donato, Milan, Italy; ^3^ Department of Cardiothoracic Center, Istituto Clinico Sant’Ambrogio, University of Milan, Milan, Italy

**Keywords:** aortic valve stenosis, small aortic annulus, transcatheter aortic valve replacement (TAVR), prosthesis–patient mismatch (PPM), cardio-CT

## Abstract

Transcatheter aortic valve replacement (TAVR) is a treatment of choice in patients with symptomatic severe aortic valve stenosis (AS) and intermediate-to-high surgical risk. The presence of a small aortic annulus (SAA) has been associated with a higher incidence of prosthesis–patient mismatch (PPM) when surgical aortic valve replacement (sAVR) is performed. TAVR might be a treatment option offering better hemodynamics with a lower incidence of PPM. When a severe AS with a SAA is treated, TAVR-related risk as the coronary obstruction and the annulus rupture, must be also prevented. We present a case of a TAVR in a very small aortic annulus; to our knowledge, this is the smallest native aortic annulus treated percutaneously in a tricuspid stenotic aortic valve with a Self-Expanding Transcatheter Heart Valve (THV) Acurate Neo 2.

## Introduction

Symptomatic severe aortic stenosis (AS) in patients with intermediate-to-high surgical risk is currently being treated with transcatheter aortic valve replacement (TAVR). When a surgical aortic valve replacement (sAVR) is performed, the presence of a small aortic annulus (SAA) is usually associated with higher incidence of a prosthesis–patient mismatch (PPM) (1). Multicenter registries have recently published the comparative data between different transcatheter heart valve (THV) replacements in patients with SAA, documenting low rates of clinical event, lower transvalvular gradients, and less incidence of PPM (2, 3). We present the case of a patient undergoing TAVR with a SAA. To our knowledge, this is the smallest native aortic annulus so far described and treated percutaneously in a tricuspid stenotic aortic valve using a self-expanding (SE) THV Acurate Neo 2.

## Case presentation

**Figure 1:**
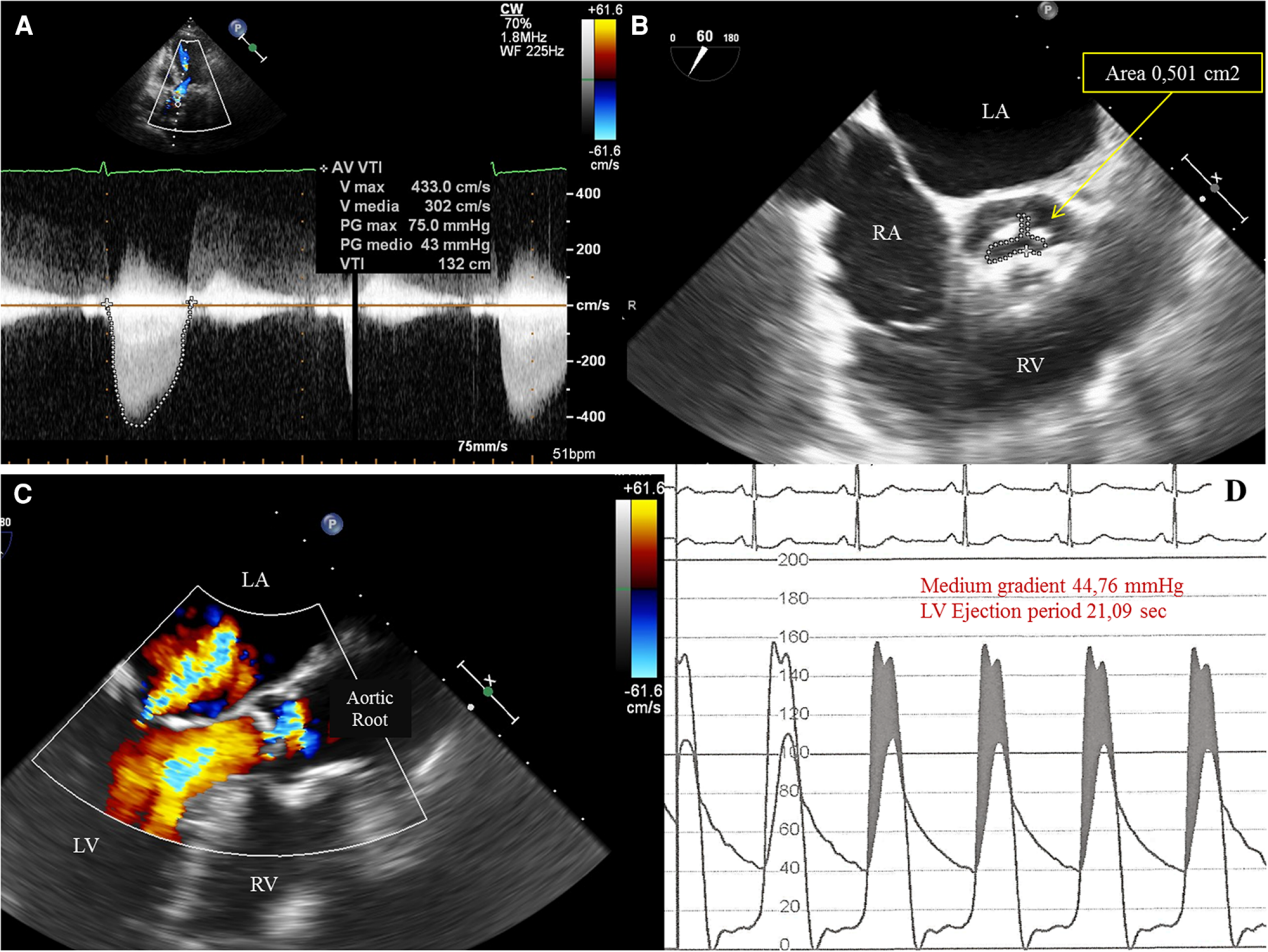
(**A**) Transthoracic echocardiography (TTE) 5-chamber view showing increased peak and medium transvalvular aortic pressure gradient of, respectively, 75 mmHg and 43 mmHg. (**B**)Transesophageal echocardiography (TEE) showing a tricuspid aortic valve with severe stenosis and a planimetric aortic valve area of 0,5 cm^2^. LA, left atrium; RA, right atrium; RV, right ventricle. (**C**) TEE showing a mild mitral valvular insufficiency with neither significant subvalvular and/or intraventricular pressure gradient nor evidence of systolic anterior motion (SAM) of the anterior leaflet of the mitral valve. (**D**) Invasive simultaneous registration of left ventricular (LV) and aortic pressures showing a significant transvalvular pressure gradient during the ventricular ejection period. LV pressure exceeds the aortic pressure (gray area, pressure gradient generated by valvular stenosis). The LV peak systolic pressure during ejection ranged between 152 to 156 mmHg and the aortic pressure between 102 to 106 mmHg. No elevated LV telediastolic pressure was documented.).

**Figure 2:**
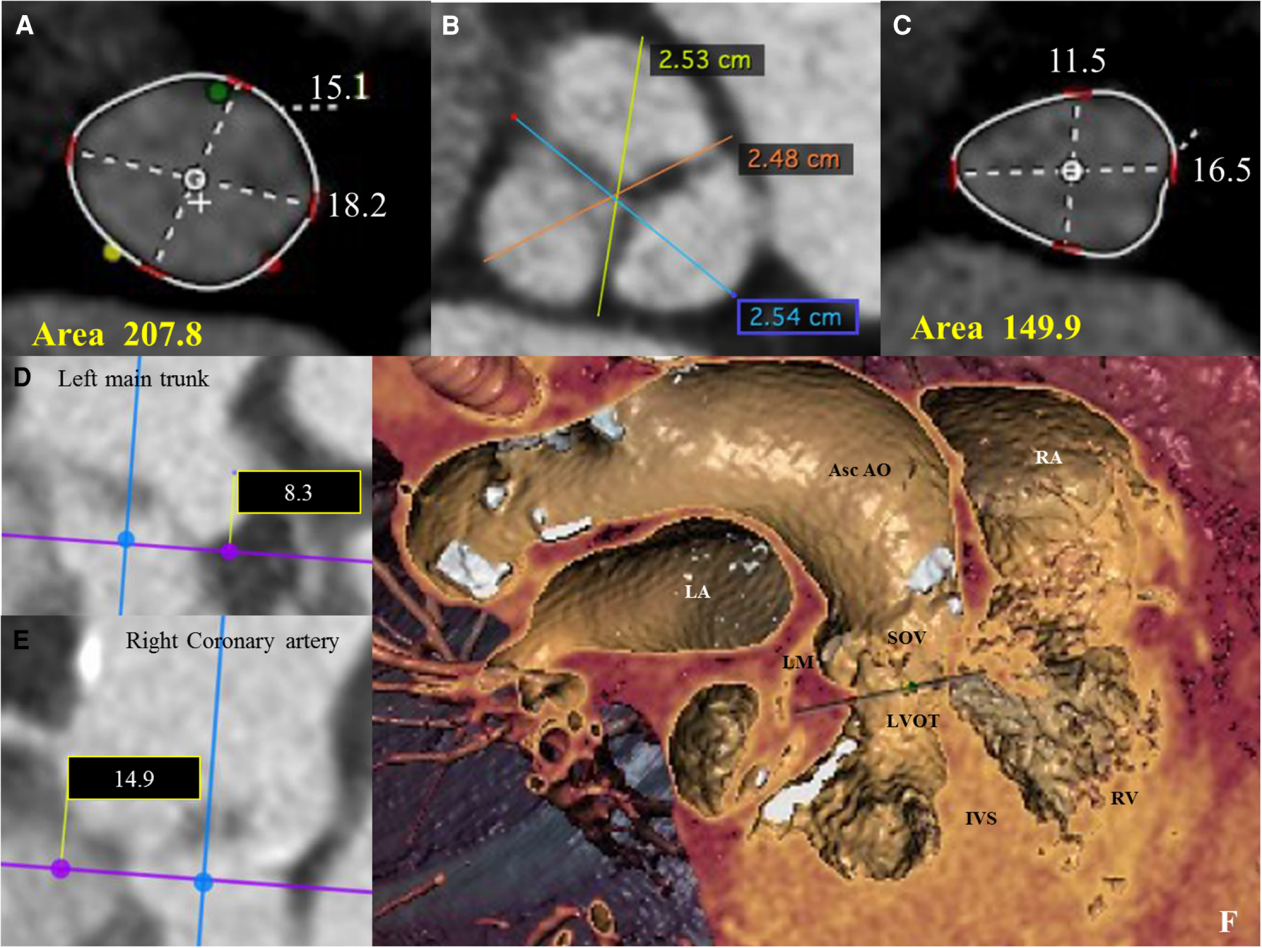
(**A**) Pre-operative thoraco-abdominal computed tomography (CT) images were analyzed using 3mensio Structural Heart software (Pie Medical Imaging, Netherlands): the aortic annulus perimeter was 51.7 mm (minor annulus diameter 15.1 mm, major annulus diameter 18.2 mm) and the area was 207,8 mm^2^. (**B**) Pre-operative thoraco-abdominal CT images showed a tricuspid aortic valve without significant valvular calcifications (images reconstructed with OsiriX DICOM Viewer Pixmeo SARL, Switzerland). (**C**) Pre-operative thoraco-abdominal CT images showed a LVOT perimeter of 45.3 mm with elliptical shape (minor annulus diameter 11.5 mm, major annulus diameter 16.5 mm) and an area of 149,9 mm^2^ (images reconstructed with 3mensio Structural Heart software). (**D** and **E**) CT images showing the distance from the virtual basal ring (VBR) to left main trunk of 8.3 mm (see **D**) and to the right coronary artery of 14.9 mm (see **E**). (**F**) Pre-operative thoraco-abdominal CT images showing the heart chambers and the ascending aorta according to the ‘double oblique virtual reality’ reconstruction with 3mensio Structural Heart software. Asc Ao, Ascending Aorta; IVS, interventricular septum; LM, left main coronary trunk; LA, left atrium; LVOT, left ventricular outflow tract; RA, right atrium; RV, right ventricle.

**Figure 3:**
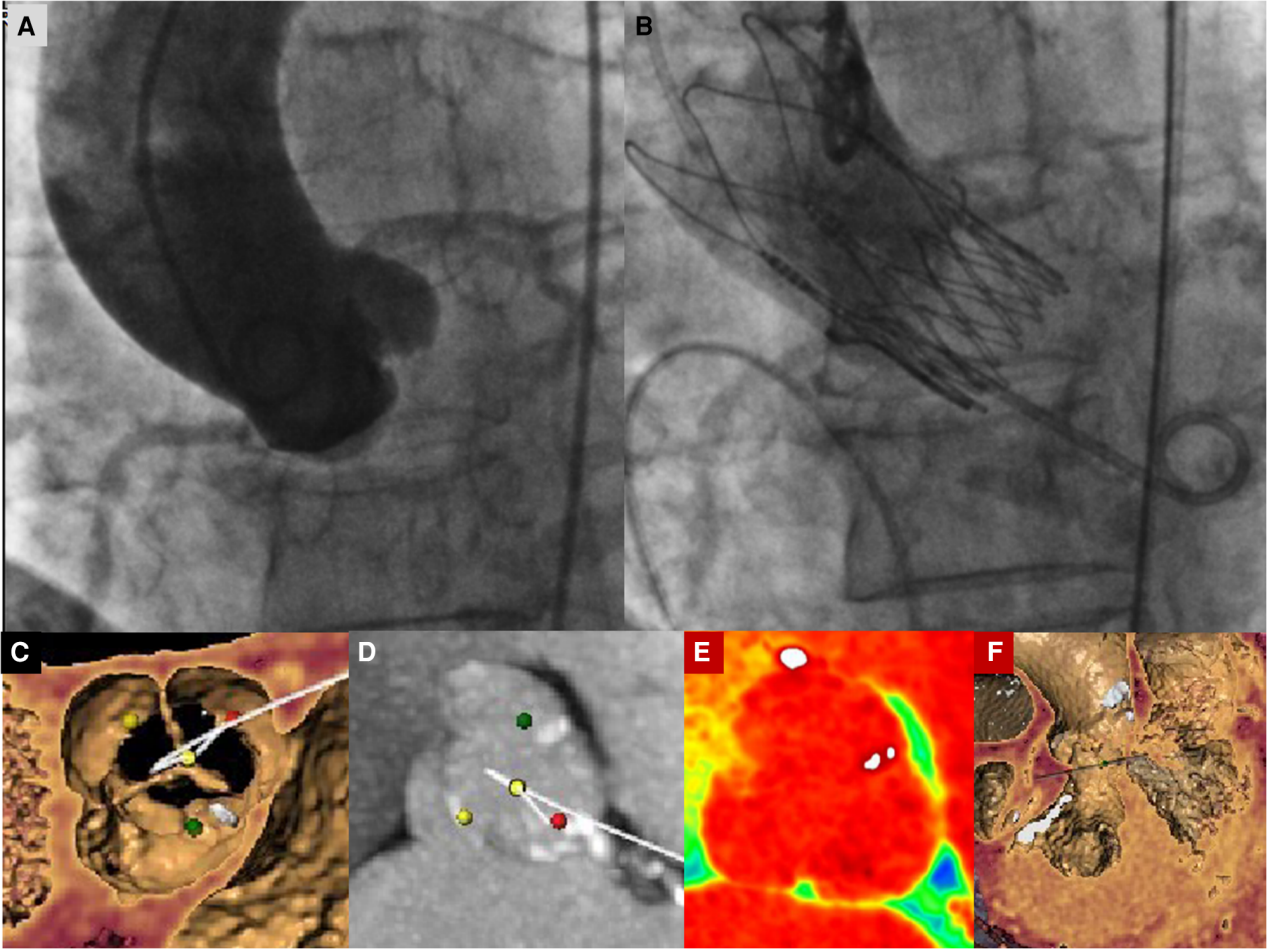
(**A**) Basal aortography in the “virtual basal plane” view (LAO 5°−10° cranial projection). (**B**) Final aortography documenting correct implantation of an Acurate Neo 2 small-size valve with patent coronary arteries and correct minimal LVOT protrusion of the THV to ensure anchoring without any trace of paravalvular leak. (**C**) Preprocedural images of CT angiography with tridimensional reconstruction of the aortic valve showing a tricuspid aortic valve. The yellow dot identifies the non-coronary cusp, the red dot identifies the left coronary cusp, and the green dot identifies the right coronary cusp. (**D–F**) Preprocedural images of CT angiography with tridimensional reconstruction of the aortic valve showing a tricuspid aortic valve without significant calcific degeneration and left and right ventricular chambers. In Panel (**D**), the yellow dot identifies the non-coronary cusp, the red dot identifies the left coronary cusp, and the green dot identifies the right coronary cusp.

We present the case of a 74-year-old female, with symptoms of dyspnea on exertion, who was evaluated by our Cardiac Team for severe aortic valve (AV) stenosis. The patient presented with European System for Cardiac Operative Risk Evaluation (Euroscore) II, which corresponds to 1.23%, and a Society of Thoracic Surgeons (STS) score of 1.443%. However, the patient suffered from significant obesity [body mass index (BMI) of approximately 33] and a chronic obstructive pulmonary disease (COPD) with emphysema and bronchiectasis, which was documented on a thoracic computed tomography (CT) scan. The body surface area (BSA) was 1.9. The transesophageal echocardiography showed a stenotic tricuspid AV (valve area of 0.51 cm^2^ and peak and mean gradients of 75 and 43 mmHg, respectively) and a normal left ventricle (LV) ejection fraction without significant intraventricular gradient (Figure 1 and Supplementary Material Table S1, Supplementary Video S1). The preoperative thoraco-abdominal computed tomography angiography (CTA) showed an AV without significant calcifications, an aortic annulus perimeter of 51.7 mm (area of 207.8 mm^2^), and a left ventricular outflow tract (LVOT) perimeter of 45.3 mm with an elliptical shape (area of 149.9 mm^2^) (Figure 2 and Supplementary Material Table S1). The TAVR procedure was performed “off-label” by implanting an SE THV in the supra-annular location of the leaflets (Acurate Neo 2, 23 mm, Boston Scientific, Marlborough, MA, USA), without any predilation (Supplementary Video S2). The “off-label” smallest size of Acurate Neo 2 was implanted with a 39.7%–58% oversizing corresponding to the waist and lower crown, respectively. The THV was implanted utilizing the “commissural alignment” maneuvers without any coronary wire protecting the left main trunk. In our opinion, the open-cell design of the Acurate Neo 2 and the upper crown’s gripping and catching of the native aortic valve leaflets would guarantee patency of the left main trunk. At the same time, greater distance from the THV and coronary ostia at the level of the Valsalva sinuses may be obtained. The prosthetic release of “Step 2” was performed with a slight push, obtaining an implant depth adequate to maintain greater space at the level of the Valsalva sinuses (in consideration of the distance of the left main trunk from the virtual basal ring which was 8.3 mm). Therefore, we did not perform “active” coronary protection to prevent coronary damage due to a guide-catheter dislocation during the process of the THV implantation. Based on the strategy of our group, we completed the “Phase 2” of Acurate Neo 2 deployment with rapid pacing to maximize the precision of the THV implantation (Supplementary Video S3). The final angiography scan showed a good result without any significant residual transvalvular gradient or paravalvular leak, and with an adequate expansion of the metallic frame of the THV (Figure 3 and Supplementary Video S4). The ECG result after the THV implantation showed a delayed intraventricular conduction that completely normalized on discharge 5 days later. We proposed that the patient undergo a short period of dual antiplatelet therapy (DAPT). The postprocedural transthoracic echocardiographic (TTE) test highlighted the correct positioning of the bioprosthesis without significant protrusion in the LVOT and without any interference with the mitral valve apparatus. The TTE conducted before discharge and 30 days after the procedure showed a mean transvalvular gradient of 10 mmHg with preserved LV function and without any intraventricular pressure gradient.

## Discussion

When a severe aortic stenosis with a SAA is treated, any TAVR-related risk, such as the PPM, the coronary obstruction, the annulus rupture, and the intraventricular conduction disturbances requiring a pacemaker implantation, must be prevented. A SAA is defined as an annular area of <400–430 mm^2^ on computed tomography; these thresholds were selected on the basis of different studies. The TAVI-SMALL registry recently demonstrated similar PPM rates with three SE THV platforms (Evolut/Pro, Acurate, and Portico) in patients with a small annulus (3). A substudy of patients with a SAA from the Japanese TAVR registry compared hemodynamics in those who received a 20-mm vs. a 23-mm Sapien XT THV (Edwards Lifesciences). The postprocedural rate of moderate or severe PPM after TAVR was 32% and 8% for the 20-mm and 23-mm THV, respectively (4). A retrospective multicenter study collected data from 8,411 consecutive patients with a SAA (<400 mm^2^) treated with TAVR. Moderate and, particularly, severe PPM was less frequent after implantation of the SE (supra- and intra-annular) THV compared with the balloon-expandable (BE) (intra-annular) or mechanically expanding (infra-annular) devices (5). To optimize the TAVR procedure and to prevent the higher risk of the complications, the following were the strategies and procedural decisions to consider: (a) choice between a SE and BE THV; (b) choice between a SE THV with intra-annular or supra-annular location of the leaflets; (c) implanting the THV with the commissural alignment maneuver to prevent coronary occlusion; and (d) avoid pre- and postdilatation, if possible.

The following are the main clinical, technical, and procedural considerations:

1. According to recommendations, the minimum perimeter of 56.5 mm and the minimum area of 254 mm^2^ represent the lower limits of the sizing guidelines for THV currently available.
2. We used a SE valve with supra-annular location of the leaflets that offers a potential advantage over BE valves and intra-annular SE valves, ensuring a larger effective area of the orifice.
3. We avoided pre- and postdilatation to reduce the risk of annular damage and/or rupture.
4. The precision of the THV implantation (with minimal protrusion in LVOT) was maximized when the rapid pacing during Phase 2 of the valve deployment was utilized.
5. A short period of DAPT can be considered in order to prevent a potential increase of thromboembolic events secondary to small aortic valve area.
6. In our opinion, the main goal is to prevent high implantation and commissural misalignment in order to facilitate the subsequent coronary percutaneous interventions, if needed. Even prophylactic coronary stenting (“chimney fashion”) should be avoided unless absolutely necessary. These technical considerations also apply to allow a subsequent valve-in-valve (VIV) procedure, if indicated.

To our knowledge, this is the smallest native aortic annulus so far described and treated percutaneously in a tricuspid stenotic aortic valve using a self-expanding THV Acurate Neo 2.

## Data Availability

The original contributions presented in the study are included in the article/Supplementary Material, further inquiries can be directed to the corresponding author.

## Supplementary material

Supplementary Video S1 Preoperative transesophageal echocardiography (TEE) (short-axis view) confirmed a severe aortic stenosis with fibrotic degeneration of the free margins of the tricuspid aortic valve. The significant thickening at the points of leaflet coaptation (nodules of Arantius) also may be identified.

Supplementary Video S2 Basal aortography in the ‘virtual basal plane' view (‘co-planar’ view) (5° left anterior oblique (LAO) and 13° cranial projection). The sinus of Valsalva and the ascending aorta are of normal size. Diffuse calcifications of the mitral annulus are also visible.

Supplementary Video S3 After complete opening of the ‘upper crown' and the ‘axial stabilization arches’ (phase 1 of the THV deployment), we completed the phase 2 deployment under rapid pacing (180 bpm) in order to maximize the precision of THV implantation (Acurate Neo 2).

Supplementary Video S4 Final aortography documented the correct ACURATE NEO 2 valve implantation with patent coronary arteries without paravalvular leak and abolition of transvalvular pressure gradient.

## References

[1] Pibarot P Dumesnil JG. Prosthetic heart valves: selection of the optimal prosthesis and long-term management. Circulation. (2009) 119:1034–48. 10.1161/CIRCULATIONAHA.108.778886 19237674

[2] Victor M Kim WK Abumayyaleh M Walther T Moellmann H Schaefer U Short-term outcome and hemodynamic performance of next-generation self-expanding versus balloon-expandable transcatheter aortic valves in patients with small aortic annulus: a multicenter propensity-matched comparison. Circ Cardiovasc Interv. (2017) 10:e005013. 10.1161/CIRCINTERVENTIONS.117.005013 28951395

[3] Regazzoli D Chiarito M Cannata F Pagnesi M Miura M Ziviello F Transcatheter self-expandable valve implantation for aortic stenosis in small aortic annuli. JACC Cardiovasc Interv. (2020) 13:196–206. 10.1016/j.jcin.2019.08.041 31883714

[4] Miyasaka M Tada N Taguri M Kato S Enta Y Otomo T Incidence, predictors, and clinical impact of prosthesis–patient mismatch following transcatheter aortic valve replacement in Asian patients: the OCEAN-TAVI registry. J Am Coll Cardiol Intv. (2018) 11(8):771–80. 10.1016/j.jcin.2018.01.273 29673509

[5] Voigtländer L Kim WK Mauri V Gobling A Renker M Sugiura A Transcatheter aortic valve implantation in patients with a small aortic annulus: performance of supra-, intra- and infra-annular transcatheter heart valves. Clin Res Cardiol. (2021) 110(12):1957–66. 10.1007/s00392-021-01918-8 34387736 PMC8639544

