# Supplementary material for: Case report: Self-expanding transcatheter valve implantation (Acurate Neo 2) in a very small native aortic annulus
Source: Front Cardiovasc Med. 2023 Sep 15;10:1195486. doi: 10.3389/fcvm.2023.1195486 (PMC10545879; doi:10.3389/fcvm.2023.1195486)
Supplement: Supplementary file 1 [file Table1.docx]

**Table 1**

**Pre-operative transesophageal echocardiography (TEE) and thoraco-abdominal computed tomography (CT)**

| **Pre-operative transesophageal echocardiography (TEE)** | |
| --- | --- |
| Valve anatomy | Tricuspid |
| TEE AVA (cm2) | 0.6 |
| TEE planimetric AVA (cm2) | 0.51 |
| TEE transvalvular gradient peak/mean (mmHg) | 75/43 |
| TEE aortic annulus (mm) | 18 |
| **Pre-operative thoraco-abdominal computed tomography (CT)** | |
| CT aortic annulus area (mm2) | 207.8 |
| CT aortic annulus perimeter (mm) | 51.7 |
| CT aortic annulus minor diameter (mm) | 15.1 |
| CT aortic annulus major diameter (mm) | 18.2 |
| CT aortic annulus perimeter derived diameter (mm) | 16.4 |
| CT aortic annulus area derived diameter (mm) | 16.3 |
| CT LVOT area (mm2) | 149.9 |
| CT LVOT perimeter (mm) | 45.3 |
| CT LVOT minor diameter (mm) | 11.5 |
| CT LVOT major diameter (mm) | 16.5 |
| CT LVOT perimeter derived diameter (mm) | 14.4 |
| CT LVOT area derived diameter (mm) | 13.8 |
| Distance to left main trunk (mm) | 8.3 |
| Distance to right coronary artery (mm) | 14.9 |

AVA, aortic valve area; CT, computed tomography; LVOT, left ventricle outflow tract; TEE, transesophageal echocardiogram
